# Hepatoprotective potential of isoquercitrin against type 2 diabetes-induced hepatic injury in rats

**DOI:** 10.18632/oncotarget.21074

**Published:** 2017-09-16

**Authors:** Xiao-Li Huang, Yang He, Li-Li Ji, Kai-Yu Wang, Yi-Li Wang, De-Fang Chen, Yi Geng, Ping OuYang, Wei-Min Lai

**Affiliations:** ^1^ Department of Basic Veterinary, College of Veterinary Medicine, Key Laboratory of Animal Disease and Human Health of Sichuan Province, Sichuan Agricultural University, Wenjiang, Sichuan, P.R. China; ^2^ Department of Aquaculture, College of Animal Science & Technology, Sichuan Agricultural University, Wenjiang, Sichuan, P. R. China; ^3^ Meat Processing Key Laboratory of Sichuan Province, Chengdu University, Chengdu, Sichuan, P.R. China; ^4^ Sichuan Tiantian Biotechnology Application Ltd, Chengdu, Sichuan, P.R. China

**Keywords:** type 2 diabetes mellitus, non-alcoholic fatty liver disease, isoquercitrin, DPP-IV

## Abstract

Non-alcoholic fatty liver disease is a main complication of type 2 diabetes. Isoquercitrin are employed for antidiabetic therapies, but the effects on liver function and the hepatocytes are unclear. The aim of this study was to investigate the effects of isoquercitrin on the T2DM-induced hepatic injury in rats. Isoquercitrin (10 mg/kg/d, 30 mg/kg/d), sitagliptin phosphate (10 mg/kg/d) was given orally for 21 days. The administration of isoquercitrin at 10 mg/kg/d and 30 mg/kg/d showed a dose dependent. Compare to the negative control (treated with saline), rats medicated with isoquercitrin (30 mg/kg/d) and sitagliptin phosphate (10 mg/kg/d) improved the clinical symptoms, FBG and glucose tolerance, reduced serum ALT, AST and IR, but increased TP, Alb, SOD, GSH, MDA, HDL-C, INS and GLP-1. On histology, Rats of these to groups presented nearly normal liver tissue and Langerhans, degeneration, necrosis and apoptosis were markedly reduced. Instead, hepatocytes showed regenerate. These two groups also showed significant increase in mRNA expression of PKA, AKT, PKCa, InsR and PI3K, and a decrease in DPP-IV mRNA level. These results indicated that treatment with isoquercitrin protects against hepatic injury by T2DM.

## INTRODUCTION

Diabetes mellitus is a chronic metabolic disorder, characterized by hyperglycemia (fasting state>7 mmol/L). Type 2 diabetes mellitus (T2DM) is the most prevalent form of diabetes, accounts for 90% of the patients. The mechanisms of T2DM are the insulin resistance (IR) of liver and muscle. Thus nearly all T2DM patients manifested severe hepatic steatosis [1].

Non-alcoholic fatty liver disease (NAFLD) is a clinicopathologic syndrome, recognized as one of the most common causes of liver damage, including hepatic steatosis [2]. Alterations in hepatic metabolism lead to overproduction of glucose and lipids, which in turn abet development of glucose intolerance and dyslipidemias to induce T2DM [3]. Patients with NAFLD increased about 5-fold of the incidence of T2DM [4]. While T2DM are the strongest predictors of NAFLD, more than 70% patients suffered NAFLD [5].

Isoquercitrin (quercetin-3-O-b-D-glucopyranoside, C_20_H_21_O_12_) is a wildly existed natural flavonoids in plants [6]. It possess various pharmacological activities, such as antioxidant [7], anti-inflammatory [8], anti-cancer [9]. Previous research has reported that isoquercitrin improved the type 2 diabetes by preventing the inactivation of glucagonlike peptide-1 (GLP-1) as a dipeptidyl peptidase-IV(DPP-IV) inhibitor [10]. And isoquercitrin protected H4IIE cells from liquid accumulation by activating the AMP–activated protein kinase (AMPK) signal pathway [11].

Thus, we hypothesis that isoquercitrin may also target the hepatocytes function in the course of therapy for T2DM. The aim of this study was to investigate the hepatoprotective potential of isoquercitrin by clinical symptoms observation, blood glucose level and biochemical indexes monitoring, MTT assay, histological analysis, flow cytometry assays and qRT-PCR analysis.

## RESULTS

### Isoquercitrin improved the clinical symptoms

To observe the effect of isoquercitrin on the improvement of clinical symptoms, the urinary output, water intake, feed intake and body weight were monitored. Results showed that isoquercitrin improved the clinical symptoms, even though the effects were not as good as sitagliptin phosphate. Rats of negative control group showed polyphagia, polydipsia and polyuria, with low spirits and yellow coat color. In comparison, animals treated with isoquercitrin and sitagliptin phosphate reduced the water and feed intake as well as the urinary output.

For average weight loss, the negative groups lost weight significantly (p<0.01) in 21st dpt of treatment. While the weight loss of rats treated with isoquercitrin slowed down, ranging from 12.39 g to 9.91 g during the 1st week, 10.54 g to 6.73 g during the 2nd week, then 9.77 g to 2.08 g in the 3rd week. And the weight of the positive control group showed stable, and gained weight at the end of the trial period (Table 1). No death was found during the experiment.

**Table 1:** Body weight after a period of treatment with isoquercitrin

Item	days	Low Dose	High Dose	Negative Control	Positive Control
Body weight(g)	0	253.39±19.01	236.92±17.99	246.91±23.27	232.89±16.00
Weight variation (compared with day 0)	7	-12.39±0.28^AB^	-9.91±0.90 ^AB^	-17.30±1.71	-7.25±0.82^A^
	14	-10.54±0.53^AB^	-6.73±1.24 ^AB^	-17.14±1.36	-4.58±0.78^A^
	21	-9.77±0.47^AB^	-2.08±1.97 ^AB^	-15.78±1.10	7±2.45^A^

Note, A means extremely significant different with negative control, B means extremely significant difference with positive control.

### Isoquercitrin reduced FBG and improved glucose tolerance

**Figure 1:**
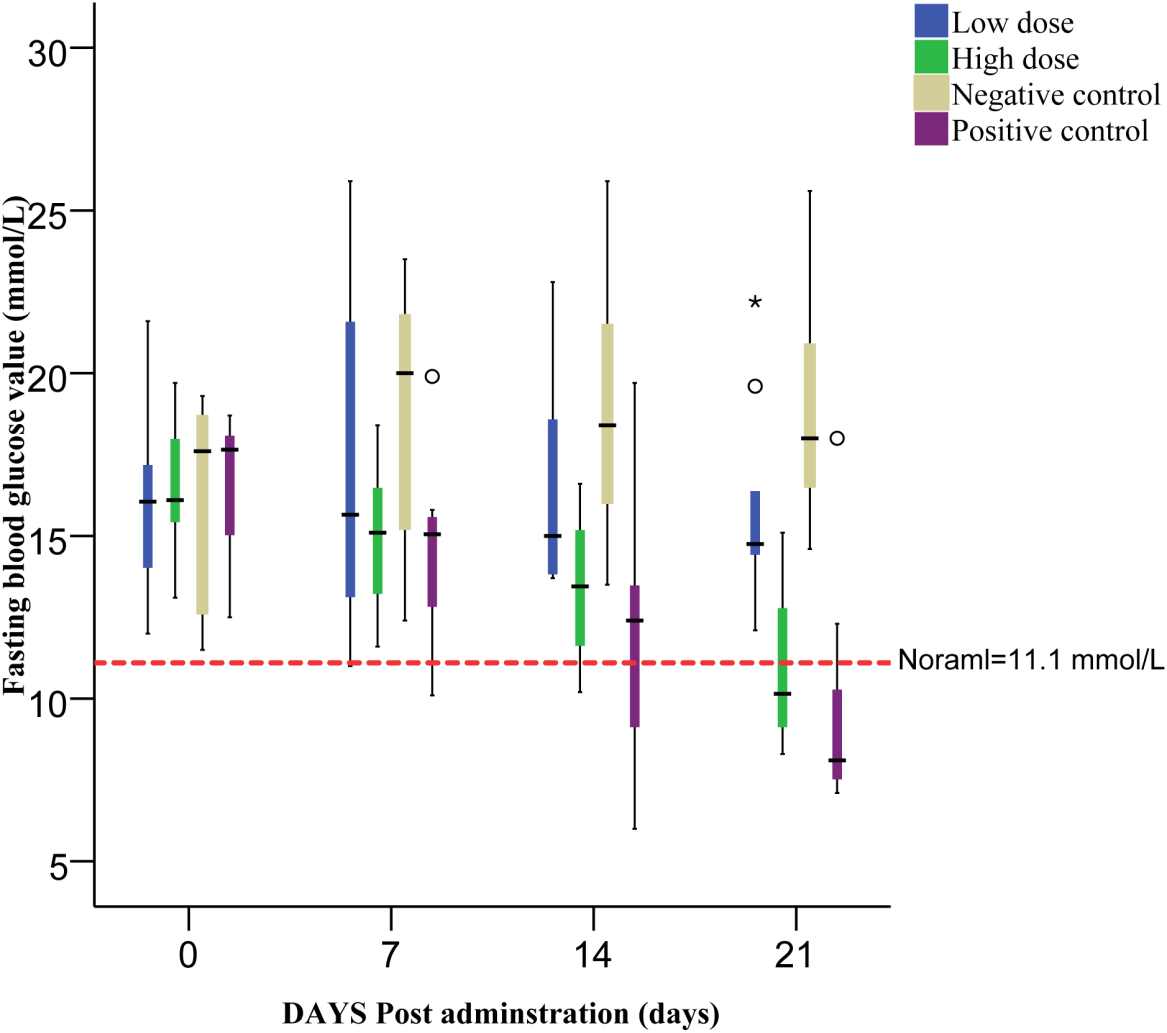
The fasting blood value of all groups monitored before each administration

To investigate the effect of isoquercitrin on therapy for blood glucose in T2DM, FBG and glucose tolerance were monitored. Results showed that the FBG in the group medicated by sitagliptin phosphate decreased mostly from 16.73 down to 9.53 mmol/L at 21 dpt, followed by the high and low dose group of isoquercitrin treatment. In contrast, rats of negative group exhibited an increase trend from 16.27 mmol/L at 1st dpt up to 19.04 mmol/L at 21st dpt (Figure 1).

**Figure 2:**
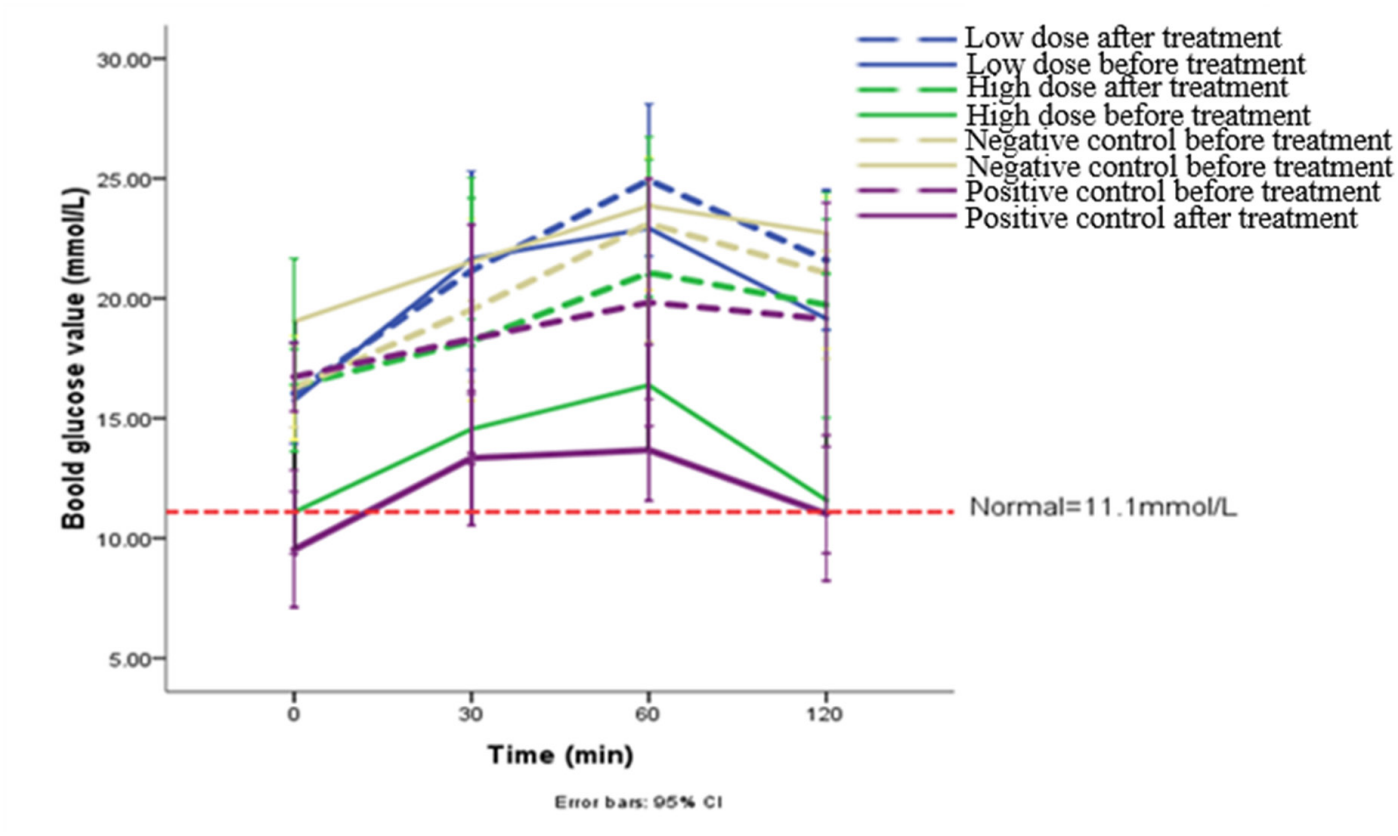
The results of OGTT before and 21 days later each administration

OGTT results showed that the blood glucose increased in all groups before treatment. The highest glucose level appeared after 60 min of administration, about 3.07-8.89 mmol/L and 18.35%-55.45% rise. 21 days the treatment, rats of high dose group and positive control group exhibited a relatively stable blood glucose value, and recovered to normal at 120 min (Figure 2)

### Isoquercitrin played a role in hepatoprotection

To explore the hepatoprotective potential of isoquercitrin, blood biochemical analysis was performed. Results showed that (Table 2).

**Table 2:** Results of rat's blood biochemical index in different groups

Item		Low dose	High dose	Negative control	Positive control
Liver function	ALP(king unit/gport)	31.86±0.40	28.72±0.99	31.11±0.55	30.41±0.77
	AST(U/gport)	156.64±5.55^AB^	142.21±3.46^Ab^	164.59±2.73	136.51±3.29^A^
	ALT(U/gport)	44.21±1.38^B^	32.47±2.33^A^	47.17±1.69	31.25±1.54^A^
	TP(mg/mL)	48.03±0.45^aB^	52.99±0.18^Ab^	46.96±0.31	49.75±0.08^A^
	Alb(g/L)	20.96±0.25^AB^	25.75±0.74^Ab^	17.94±0.48	28.95±0.70^A^
Anti-oxidase activity	GSH(μmol/L)	5.52±0.24^AB^	6.71±0.17^AB^	2.65±0.15	9.14±0.20^A^
	SOD(U/mL)	172.21± 2.88^B^	185.81±10.01^aB^	160.92±5.81	215.01±3.83^A^
	MDA(nmol/ mL)	30.47± 2.20^B^	28.45±2.91^B^	30.68±1.33	21.67±2.11^a^
Lipid metabolism	LDL-C(mmol/L)	2.46±0.04	2.36±0.11	2.62±0.09	2.18±0.15^A^
	HDL-C(mmol/L)	0.32±0.02^aB^	0.34±0.03^AB^	0.24±0.01	0.23±0.02
	T-CHO(mmol/L)	2.22±0.13	2.11±0.04	2.30±0.05	2.09±0.05
	TG(mmol/L)	1.21±0.03	1.21±0.06	1.22±0.09	1.24±0.04
IR	INS(ng/mL)	0.55± 0.10^A^	0.74±0.10^a^	0.45±0.03	0.68± 0.11
	GLP-1(ng/mL)	0.87± 0.05^B^	1.22±0.22 ^a^	0.62± 0.09	1.18±0.12^a^
	HOMA-IR	7.80±0.31^b^	6.99±0.97^a^	7.98±0.52	6.48±0.75^a^

Note, a and A means significant different (p<0.05) or extremely significant different (p<0.01) with negative control; b and B means significant different (p<0.05) or extremely significant different (p<0.01) with positive control.

Comparing to the negative control group, rats treated with medicine decreased in ALT and AST significantly, while increased in TP and Alb significantly. Rats treated with isoquercitrin showed a dose-dependent decrease of AST and ALT and increase of Alb and TP. The high dose of isoquercitrin showed a better effect on TP (52.99±0.18 mg/mL) than that of sitagliptin phosphate (49.75±0.08 mg/mL), (p<0.05).

Anti-oxidase activities showed that the isoquercitrin increased the activities of SOD and GSH and decrease MDA, significantly. Lipid metabolism results showed that HDL-C increased in the groups medicated with isoquercitrin, significantly higher than that of the positive and negative groups, even though no obverse effects on LDL-C, T-CHO and TG.

Insulin resistance index showed that INS and GLP-1 were increased in all the medicated groups after the treatment. And the high dose group showed the highest INS (0.74±0.10 ng/mL) and GLP (1.22±0.22 ng/mL). HOMA-IR of the medicated groups were significantly decreased. The isoquercitrin treated groups displayed a dose-dependent decrease of HOMA-IR, with no significant difference to that of the positive group.

### Isoquercitrin promoted the glucose consumption

To evaluate the effects of isoquercitrin on glucose consumption of hepatocytes, an *in vitro* research with HepG2 was applied. The glucose consumptions results showed that isoquercitrin promoted the glucose consumption in a dose dependent pattern (Table 3). Among the concentrations, 0.125 mg/mL isoquercitrin was found to consume a volume of about 2.707 ± 0.076 mmol/L within 24 h, followed by the concentration of 0.063 mg/mL and 0.031 mg/mL (2.645±0.065 and 2.267±0.102, respectively). Results of MTT showed that isoquercitrin treatment at concentration between 0.031 to 0.125 mg/mL had no cytotoxic nor proliferative effect on HepG2. Since isoquercitrin showed no effects on the proliferation of HepG2, we infer that the glucose was consumed by the increased function of HepG2.

**Table 3:** The effect of isoquercetin on the glucose consumption of HepG2 cell

Group	Dose(mg/mL)	Glucose consumption (mmol/L)	Glucose consumption correction GC/MTT(mmol/L)
PBS	--	1.902±0.094	1.886±0.095
isoquercitrin	0.031	2.377±0.088	2.267±0.102^A^
	0.063	2.677±0.065	2.645±0.065^A^
	0.125	2.798±0.079	2.707±0.076^A^

Note, A means extremely significant difference (p<0.01).

### Isoquercitrin improvement of hepatocyte and islet cell recovery

**Figure 3:**
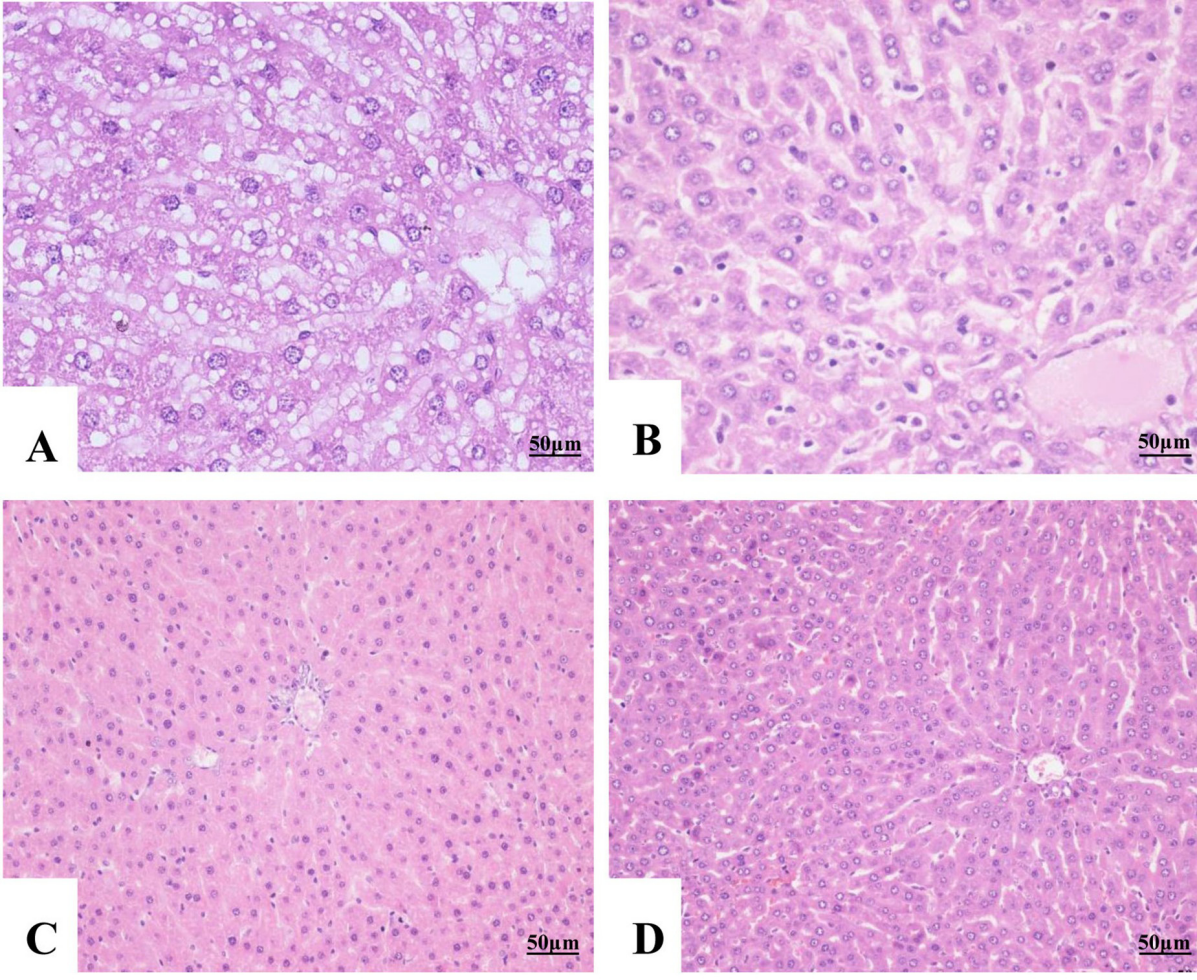
Histopathological changes of liver tissue (H&E staining, ×400) **(A)** Negative control, vacuolar degeneration and necrosis were observed in the hepatocytes. **(B)** Low dose, widespread granular degeneration was observed in the hepatocytes. **(C)** High dose, mild swelling was observed in the hepatocytes with arranged in cords. **(D)** Positive control, hepatocytes showed normal appearance and regeneration.

To investigate the hepatoprotective effect of isoquercitrin against degeneration, histopathological changes of liver and islet were observed. Results of liver tissue showed that: negative group presented loss of hepatocytes architecture, hepatic necrosis associated with vacuolar or balloon degeneration of hepatocytes. While in the high dose group and positive group, most of the cells tend to be normal. Hepatocytes of these two groups exhibited moderate swollen, and arranged in cords (Figure 3).

**Figure 4:**
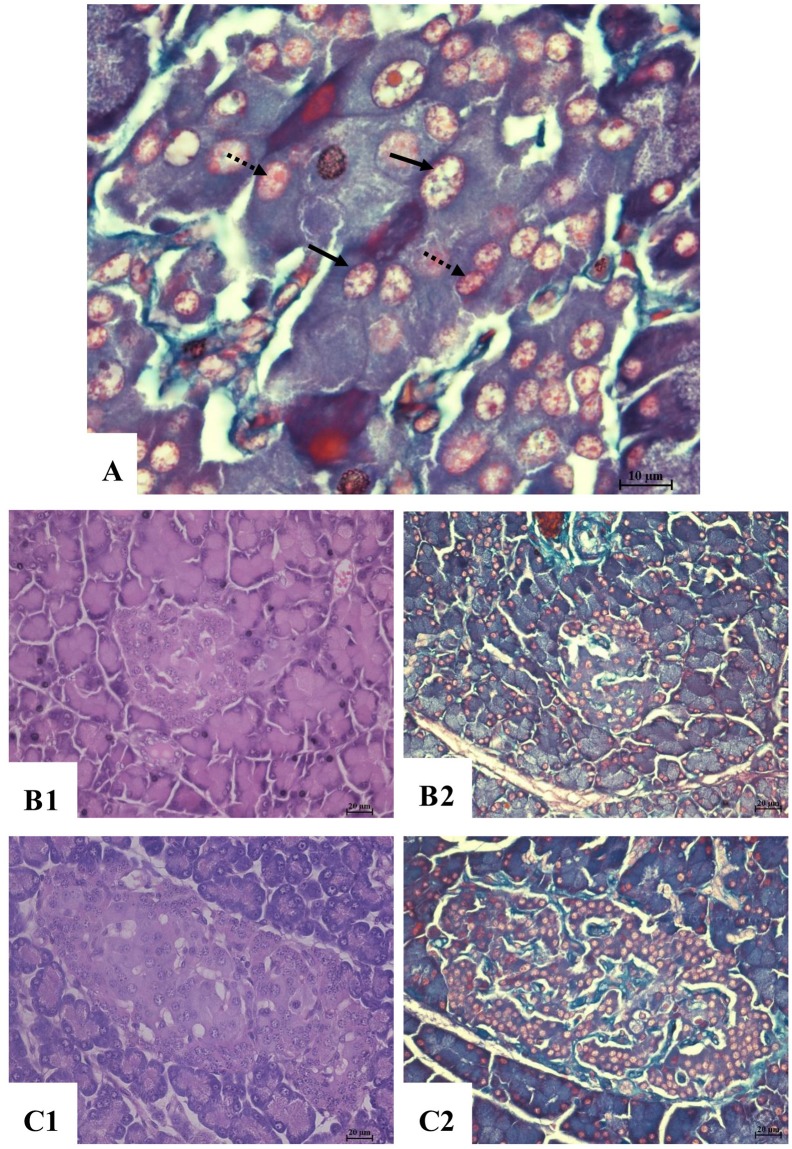
Histopathological changes of pancreatic islet tissue **(A)** aldehyde fuchsin staining ×1000, α cell (arrow) red orange colored granules in the cytoplasm, with a light orange colored nucleus; β cell (dot arrow), purplish red colored granules in the cytoplasm, with bright purple colored nucleus. **(B1)** Negative control, H&E, ×400. **(B2)** Negative control aldehyde fuchsin staining, ×400. **(C1)** High dose, H&E, ×400. **(C2)** High dose, aldehyde fuchsin staining, ×400.

Results of pancreatic islet tissue showed that(Figure 4): the negative control rats exhibited pancreatic atrophy and necrosis with disrupted architecture of Langerhans. While in the high dose group and positive group, the islet structure is nearly normal, with only to show a small number of cell degeneration or necrosis. Distinguishing the islet cells by aldehyde fuchsin showed that the negative groups presented the least number of pancreatic cells, especially the β-cells. While the high dose group and positive group exhibited much more β-cells.

### Isoquercitrin suppressed the apoptosis and promotes regeneration of hepatocytes

**Figure 5:**
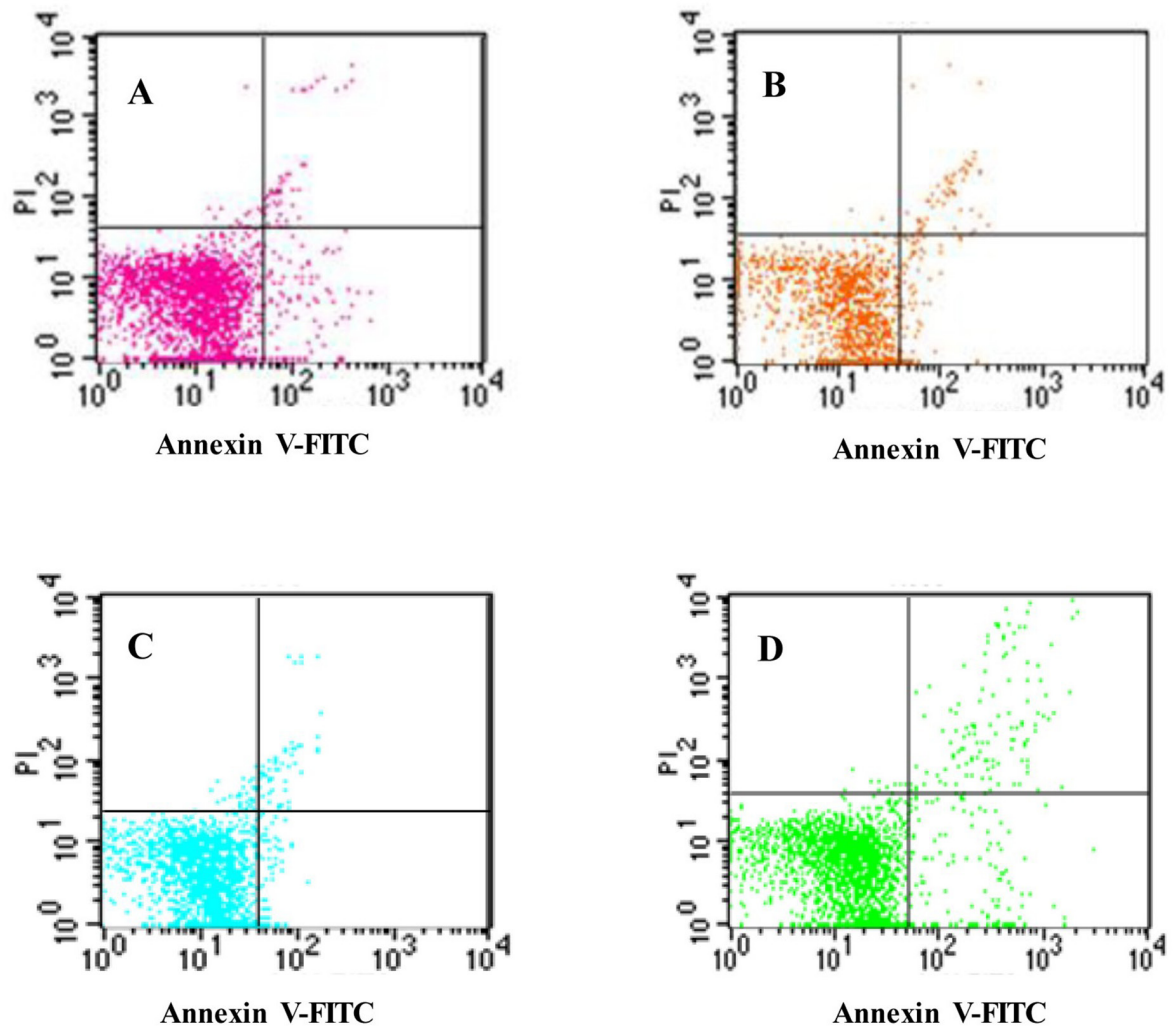
Cell apoptosis in liver cells of rats **(A)** Low does group, **(B)** High does group, **(C)** Positive control group, **(D)** Negative control group.

**Figure 6:**
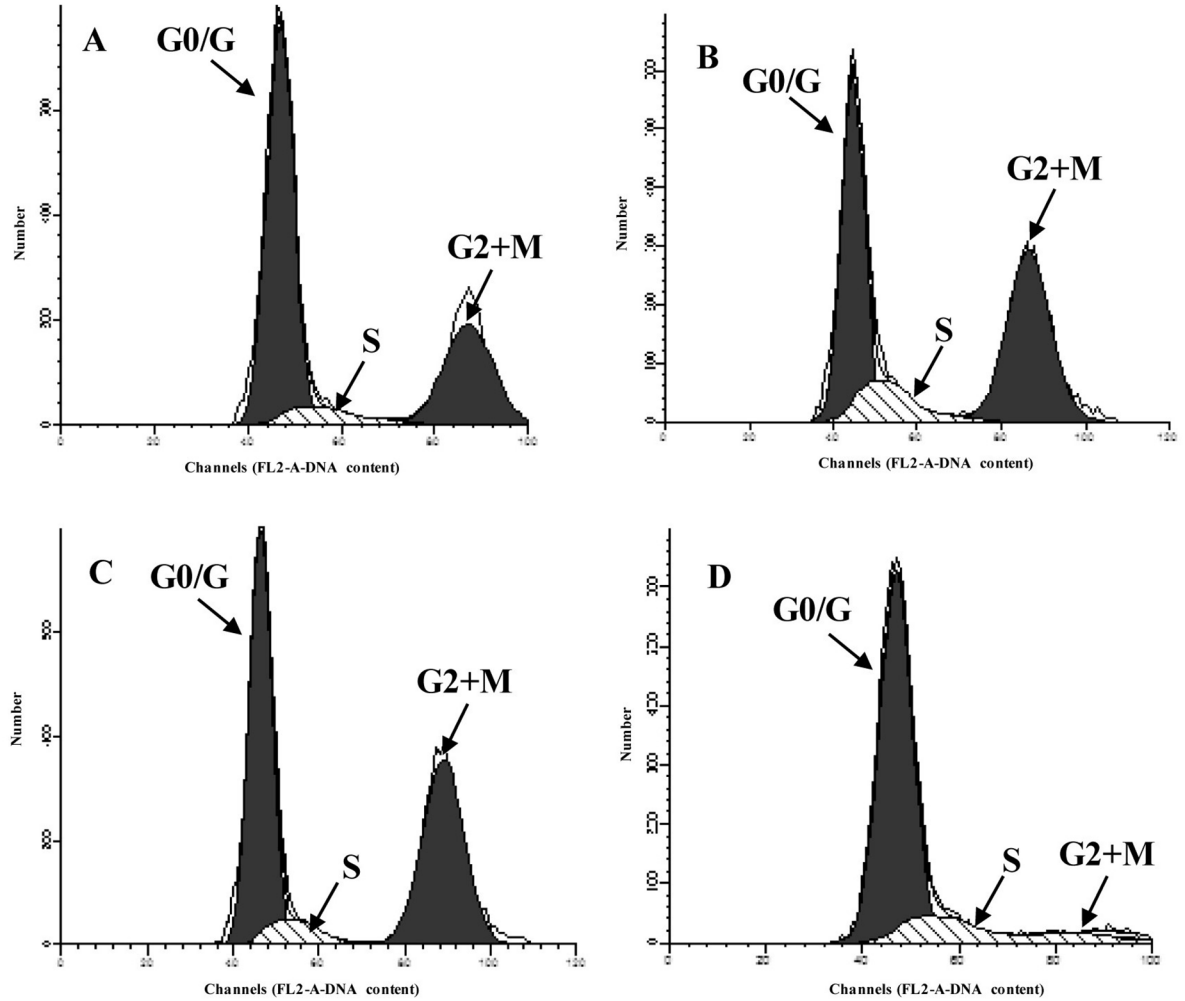
The cell cycle of liver by flow cytometry **(A)** Low does group, **(B)** High does group, **(C)** Positive control group, **(D)** Negative control group.

To further confirm the role of isoquercitrin in diabetic-induced hepatocytes, FCM was performed for apoptosis and cell cycle phase distribution. Results showed that isoquercitrin suppressed the apoptosis, in a dose-dependent pattern (Table 4, Figure 5). In the low dose group, the number of late apoptotic cells decreased significantly compared with that in the negative control (p<0.01). While the high dose group showed a significant decrease in both the early and late apoptotic cells (p<0.01). In accordance with the apoptotic results, the cell cycle phase distribution showed dose dependent decreases in G0/G1 (p<0.01) and S (p<0.05) and increases in G2+M (p<0.01), compared with that of the negative control. No significant differences were appeared between the high dose group and the positive group (Table 5, Figure 6).

**Table 4:** The apoptotic rate of rat's hepatocytes

Apoptosis rate	Low does group	High does group	Negative control group	Positive control group
Early apoptosis rate	4.48±0.08^B^	2.35±0.08^Ab^	4.13±0.42	1.70±0.01^A^
Late apoptosis rate	4.47±0.01^Ab^	3.16±0.07^A^	6.29±0.63	3.69±0.12^A^
Apoptosis rate	9.22±0.35^aB^	5.51±0.15^A^	10.42±0.23	5.39±0.12^A^

Note, a and A means significant different (p<0.05) or extremely significant different (p<0.01) with negative control; b and B means significant different (p<0.05) or extremely significant different (p<0.01) with positive control.

**Table 5:** The hepatic cycles of rats in each group (%)

period	Low does	High does	Negative control	Positive control
G0/G1	63.64±0.87^AB^	46.72±1.59^A^	81.89±1.05	44.77±1.53^A^
S	7.25±0.79^Ab^	10.75±1.46^a^	16.20±1.05	9.44±1.18^A^
G2+M	29.1±0.08^AB^	42.53±0.32^A^	1.91±0.02	45.80±0.36^A^
PI	36.36±0.87^AB^	53.28±1.59^A^	18.11±1.05	55.23±1.53^A^

Note, Proliferation Index (PI)=[S+(G2+M)]/[(G0/G1)+S+(G2+M)]. a and A means significant different (0.01<p<0.05) or extremely significant different (p<0.01) with negative control; b and B means significant different (0.01<p<0.05) or extremely significant different (p<0.01) with positive control.

### Isoquercitrin suppressed the transcription of DPP-IV

**Figure 7:**
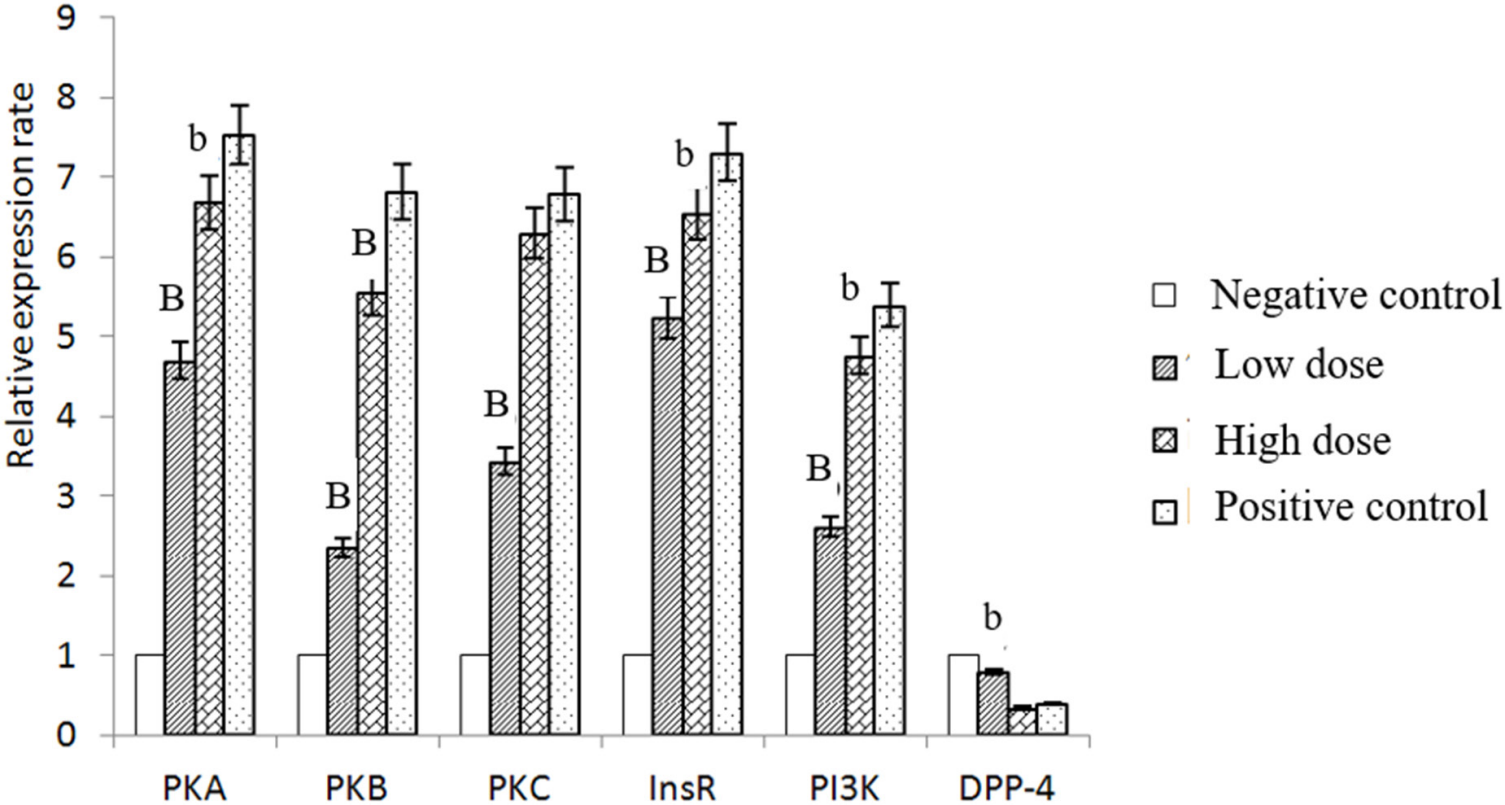
The relative expression rate of different gene in every group

To explore the molecular pathways of isoquercitrin, a qRT-PCR analysis was performed. Results showed that isoquercitrin increased the mRNA expression of PKA, AKT, PKCα, InsR and PI3K, and reducing that of DPP-IV in the positive group significantly, followed by the high dose group and the low dose group. In contrast, the mRNA level of DPP-IV showed a significant decrease, especially in the rats of high dose group (Figure 7).

## DISCUSSION

The study explores the role of isoquercitrin in hepatoprotective potential against T2DM-induced hepatotoxicity in rats. We found that a dose of 30 mg/kg/day within 21 day had therapeutic effects on hepatoprotection and Langerhans regeneration. The results showed that isoquercitrin significantly promoted the glucose consumption of hepatocytes *in vitro*. Blood biochemical index indicated that isoquercitrin improved the liver function. The histopathological changes also showed that isoquercitrin could improve hepatocytes and islet cell recovery in T2DM. Zhou [11] demonstrated that isoquercitrin could reduce the lipid accumulation in rat hepatoma H4llE cells. In the present research, hepatocytes showed an obvious improve of the vacuolar degeneration in the negative group, and nearly to normal morphology in the high dose group at 21^st^ dpt. A further sultan staining of liver tissue will perform in the following research to confirm the mechanisms of isoquercitrin for hepatoprotection.

Meanwhile, we also found that isoquercitrin promoted the proliferation of hepatocytes by increasing the cell cycle in G2+M and suppressing the apoptosis, which in contrast to the researches of Huang [12], who confirmed that isoquercitrin block the cell cycle in G1 phase in human cancer liver cells. Further researches are required to reveal these two different phenomena. Further, we found that isoquercitrin suppressed the mRNA-expression levels of DPP-IV in hepatocytes, similar to the results that isoquercitrin was an inhibitor of DPP-IV [10]. DPP-IV inhibitors are effective therapies for T2DM. They are safe, highly tolerated and body-weight neutral. However, they are highly cost for clinical use [13]. Isoquercitrin is the most abundant natural flavonoid quercetin and is ubiquitously distributed in the plant kingdom [6]. Thus, it might be a cheaper DPP-IV inhibitor for the clinical use in the future.

Hyperglycemia is one of the chief factors contribute to diabetes, which leads to 3 classical symptoms of polydipsia, polyuria and weight loss. Hyperglycemia promotes glucose enters the proximal tubules kidney and exceeds the capacity of the tubules to pump back, which resulting in an increase of osmotic effects and causing loss of water. As a consequence of dehydration and calories lost, thirst arises and weight loses [14]. Clinical observation showed that high dose of isoquercitrin relieved the clinical signs. It has been confirmed that aqueous extract of *M.oleifera* leaves has hypoglycemic properties in diabetic rats [15]. Our previous study showed that Isoquercitrin holds 41.42% of flavonoids from hydroethanolic extracts of moringa leaves (not published). Then we investigated whether isoquercitrin benefited to reduce the blood glucose. As results showed high dose of isoquercitrin reduced the fasting blood glucose concentration as well as the 2-h PG (blood glucose 2 h after an OGTT). The data are consistent with the hypothesis that isoquercitrin improved the diabetic clinical symptoms by controlling the blood glucose concentration.

AST and ALT are transaminases, which mainly exist in the hepatocytes. Thus once the hepatocytes injured, the transaminase will be released into the blood, leading to a high transaminase activity (especially AST and SLT). Alb (albumin) is mainly synthesized by the liver. The liver dysfunction, will accompanied with albumin decrease in blood [16]. The decrease of TP (total serum protein) is mainly because of the polyuria which flushes the proteins through urine. Melo [17] reported that rats in a diet of cassava leaves flour, showed a reduction in the hepatic function with ALT increased significantly, but no effects on AST and ALP and hepatocytes cytoplasm vacuolization. Our studies on the hepatic parameters exhibit a hepatoprotective potential of isoquercitrin, that the AST and ALT decreased after 21 days' therapy and increased the TP and Alb.

Previous study showed that IR is correlated with hypertriglyceridemia [18]. Hypertriglyceridemia is thought to be the main lipid abnormality in insulin resistance, which relates to the reduce of HDL-C and increase of LDL-C and TG [19]. The liver is a key site of insulin action for the synthesis and disposal of lipids. In insulin resistant states, the circulating free fatty acid (FFAs) from visceral adipose tissue increased which leads to decreased degration of apo B, thus causing an overproduction of VLDL in the liver and results in NAFLD [20]. Our data of IR showed that the model we built on rats were insulin resistant, with reducing of HDL-C and increasing of LDL-C and TG. After treated with medication, rats showed increased HDL-C and improved the insulin sensitivity with a decrease of vacuolization in the hepatocytes. However, the LDL-C and TG showed no significant difference. The reason underling needs a further research.

SOD and GSH, exist in all normal cells, and exert as important scavengers of reactive oxygen radicals to reduce the oxidative stress and the active the inflammatory mediators. The levels of MDA are often used as an indication of oxidative damage and as a marker for free radicals- induced lipid peroxidation [21]. It has been shown that SOD activity significantly reduced while the MDA contents increased in the sera and hepatic tissue in the rats with NAFLD [22]. Previous investigations have demonstrated that excess oxidative stress kills cells either by necrosis or by apoptosis [23]. In this study, we found that SOD and GSH were significantly increased, and the content of MDA decreased after medication. Further more, a significantly decrease of cell numbers undergoing apoptosis was found in liver of positive and isoquercitrin groups compare with the control group. Results indicates that the isoquercitrin may inhibit the apoptosis by antioxidation.

Previous researches have showed that the secretion of GLP-1 was reduced in patients with type 2 diabetes [24]. GLP-1 (gut hormones glucagon-like peptide-1) is derived from proglucagon through post-translational processing in enteroendocrine L cells [25, 26]. It plays a key role in stimulating insulin secretion by increasing the c-AMP levels and promoting insulin biosynthesis though PKA activation [27]. It also triggers proliferative pathway and suppress apoptosis of β cells and inhibits glucagon secretion of α-cells [28, 29]. The enzyme DPP-IV is the principal determinant of the biological half-life of intact GLP-1, which would degrade of GLP-1 within 2 minutes. Thus DPP4 inhibitors became potential antidiabetic agents. In the present research, the mRNA level of GLP increased significantly in the treatment groups, which consisted with the results of decreased DPP4. And through a further increase of transcription of PKA, PI3K/AKT and PKCα, which are believed to be pathways for anti-apoptosis, the hepatocytes metabolism capability increased and it lead the hepatic cells survived.

## MATERIALS AND METHODS

### Rats, cells and the chemicals

All experiments were conducted with the minimum number of animals and in obedience to the guidelines of the Animal Care Committee from the Sichuan Agricultural University. Male wistar rats (about 200 grams) were maintained in an enriched environment with a room-controlled temperature, 12 h light-dark cycle, food and water available *ad libitum*. HepG2 cell was a gift from Chengdu institute of biology, Chinese academy of science. Cells were maintained in RPMI-1640 or DMEM (high glucose) (Sigma, USA) containing 10% bovine calf serum (BCS) (Invitrogen, USA) and 100 U/mL penicillin-streptomycin at 37 °C with 5% CO_2_. The Isoquercitrin (98% purity) was purchased from National Institutes for food and drug control of China. Sitagliptin phosphate (100 mg/pill) was produced by Merck sharp & Dohme Italia SPA (Italy).

### Diabetes mellitus model

The T2DM model was established as described previously, with some modifies [30]. Wistar Rats were injected intraperitoneally with 40 mg/kg streptozotocin (STZ) after high-caloric diets (a total of 4297 kcal, 29.3% from fat and 42.8% from carbohydrate) for 30 days. 7 days after the injection, the blood samples were collected to evaluate blood glucose concentration by the glucose oxidase method with a blood glucometer. Only rats with a fasting blood glucose (FBG) above 7.8 mmol/L or two hours postprandial blood glucose(2HPBG) above 11.1 mmol/L were considered as T2DM and were used in the corresponding groups in the experiment.

### Isoquercitrin treatment

**Figure 8:**
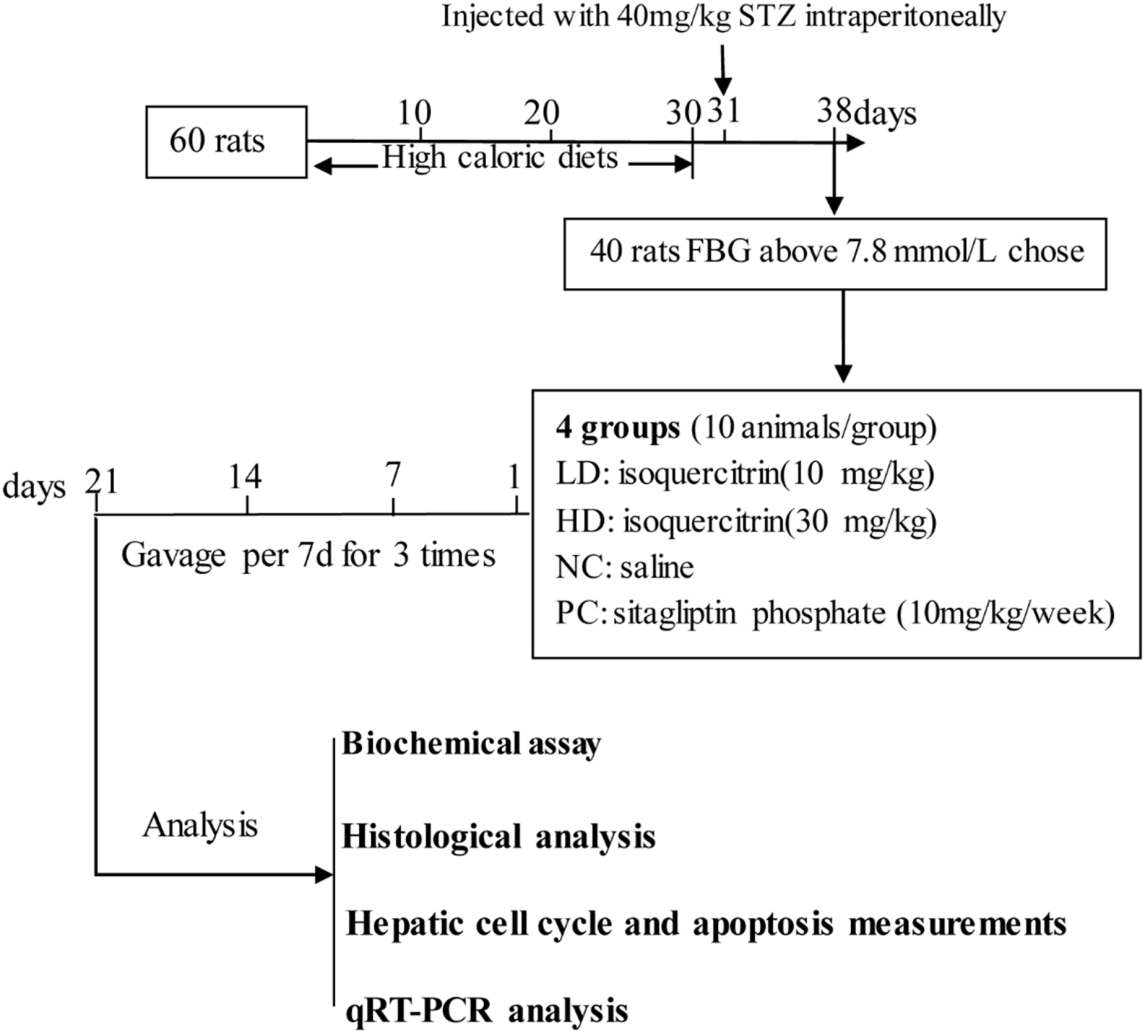
Time-line diagram for research design

The T2DM rats were randomly grouped into four different groups with 10 animals in each group, and assigned to the following treatment for 3 weeks Figure 8.

(1) The low dose group was given a gavage of isoquercitrin (10 mg/kg/day) for 21 days.

(2) The high dose group was given a gavage of isoquercitrin (30 mg/kg/day) for 21 days.

(3) The positive control group receive a gavage of sitagliptin phosphate (10 mg/kg / day) during the period [31].

(4) The negative control group received only saline for once daily during the period.

### Clinical observation

During the treatment period, drinking, eating, clinical signs and urine volume were observed daily. The body weight, water consumption, food intake was determined weekly.

### Monitor the fasting blood glucose and Oral glucose tolerance tests

During the treatment, blood was sampled from a tail vein to monitor FBG weekly. And at the 1^st^ and 21^st^ day post treatment (dpt), an Oral glucose tolerance tests (OGTT) were performed whereby overnight fasted rats received 50% glucose (5g/kg) via gavage feeding. Blood sample was collected from a tail vein at 0, 30, 60, 120 min for the determination of glucose concentrations.

### Biochemical assay

At the end of the treatment (21^st^ dpt), blood samples were collected in heparinized tubes from the eyeballs of rats after fasting for 24 h and centrifuged to obtain the plasma. Alkaline phosphatase (ALP), Aspartate aminotransferase (AST), Serum alanine aminotransferase (ALT), glutathione (GSH), Superoxide dismutase (SOD), Malondialdehyde (MDA), Total protein (TP), Albumin (Alb), High-density lipoprotein cholesterol (HDL-C), Total cholesterol (T-CHO), Triglyceride(TG) were detected using biochemical methods following the instructions of assay kits purchased from Nanjing Jiancheng Bioengineering Institute of China (ALP: Cat. No.A059-2; AST: Cat. No.C010-2; ALT: Cat. No.C009-2; GSH: Cat.No. A006-2; SOD: Cat.No.A001-1; MDA: Cat.No.A003-1 TP: Cat. No. A045-2; Alb: Cat. No. A028-1; HDL-C: Cat. No. A112-3; T-CHO: Cat. No. A111-2; TG: Cat. No. A110-1). The absorbance of ALP, AST, GSH, TP, Alb, HDL-C, T-CHO and TG were measured at 520 nm, 510 nm, 510 nm, 405 nm, 595 nm, 628 nm, 546 nm, 510 nm, 500 nm, respectively using a microtiter plate reader (Thermo, Varioskan Flash, USA).

The serum insulin (INS) and glucagon-like peptide-1 (GLP-1) were detected using enzyme linked immunosorbent assay following the instructions of reagent kits purchased from KymElisa Bioengineering Institute of China (INS: Cat. No. ISC10330EIA-2693R; GLP-1: Cat. No. KYM10330EIA-2708R). The absorbance of INS and GLP-1 were measured at 450 nm using enzyme labeling instrument (Bio-Rad, USA).

LDL-C (Low-density lipoprotein cholesterol) was calculated from the T-CHO and HDL-C: LDL-C=(T-CHO)-(HDL-C). Insulin resistance (IR) index was evaluated by the HOMA method as followed: HOMA-IR=fasting serum insulin /22.5(e ^-Ln glucose^); The fasting serum insulin is expressed in mIU/L, and glucose in mmol/L [32].

### Glucose consumption and MTT assay

HepG2 cells were analyzed. Cells were incubated to reach 80% confluence in 96- well plates. The growth medium was replaced by fresh 1640 without serum to starve the cells. 12 hours' post-starvation, cells were treated with 180 μL DMEM containing 20 μL Isoquercitrin (0.031, 0.063, 0.125, mg/mL, respectively) or PBS (as control) for 24 h. The culture medium was collected for glucose consumption testing by glucose detection kids following the instructions of reagent kits purchased from Nanjing Jiancheng Bioengineering Institute of China (Glu: Cat. NO.F006). The glucose consumptions (GC, mmol/L) = the primary concentration of glucose (G0, mmol/L) - the glucose concentration 24 h later (G24, mmol/L). After the glucose consumption assay, cells were incubated with 150 μL fresh DMEM and 50 μL MTT (2 mg/mL) for another 4 h and followed by the addition of DMSO (1ml), gentle shaking for 10 min. Results were detected at 490 nm.

### Histological investigation

At the end of the treatment, 5 rats in each group were sacrificed, then liver tissue and pancreatic islet tissue were sampled, formalin-fixed and processed in paraffin routinely. Sections (5 μm) sliced from each block were stained with hematoxylin and eosin (H&E). And the aldehyde fuchsin staining method was performed to diagnose the pancreatic islet tissue. Accordingly, 5 μm sections were oxidized by Lugol's iodine solution (20-30 min), bleaching by 2.5% sodium thiosulfate (10 sec) and washing with water (3 min) and ethanol 65% (3 times). Aldehyde fuchsin staining (80 sec), washing with ethanol 60% (3 times) and acetic acid 0.5% (15 sec) were carried out. Then followed by ponceau staining (30 sec), washing with acetic acid 0.5% (15 sec), phosphotungstic acid solution 3% (10 sec), acetic acid 0.5% (15 sec) and NaOH 0.01% (10 sec). Brilliant Green staining 2% (1 sec) and differentiation with acetic acid 0.5% were performed. Moreover, ascending percentages of ethanol, clearing, and imaging with light microscope (Olympus× 400) were carried out.

### Hepatic cell cycle and apoptosis measurements

At the 1^st^ and 21^st^ dpt, the liver of five rats in each group were sampled for determination of the cell cycle and apoptosis by flow cytometry (FCM), proposed by Cui et al [33]. Livers were immediately removed and ground to form a cell suspension that was filtered through 300-mesh nylon screen. The cells were suspended in 1×binding buffer at a concentration of 1×10^6^ cells/mL after wash the cells twice with cold phosphate buffer solution (PBS, pH 7.2-7.4).

#### Cell cycle analysis

Five hundred microliters of the solution were transferred to a 5 mL culture tube and centrifuged (500-1,000 rpm). After removing the supernatant, 5 μL 0.25%Triton X-100 and 5 μL propidium iodide were added to the tube. Then the cells were gently vortexed and incubated for 30 min at 25 °C in the dark. Finally, 500 μL PBS was added to each tube, and the contents were analyzed by FCM (BD FACS Calibur) within 45 min. Proliferation index (PI)=[S+(G2+M)]/[(G0/G1+S+(G2+M))] ×100%;

#### Apoptosis analysis

One hundred microliters of the solution were transferred to a 5 ml culture tube, then subsequently adding 5 uL of Annexin V-FITC and 5 μL of propidium iodide to the tube. The cells were gently vortexed and incubated for 15 min at room temperature (25 °C) in the dark. Four hundred microliters of 1×binding buffer was added to each tube and analyzed by FCM within 1 h.

### qRT-PCR analysis

At day 1 and 21 of the treatment, the livers from five rats in each group were stored in liquid nitrogen, respectively. Total RNA was extracted from the livers by RNA isolate (RNAiso) Plus (Takara, Japan). Then the total RNA reverse-transcribed into cDNA using a Prim-Script™ RT reagent Kit (RR047A, Takara, Japan) according to the manufactures instructions. The cDNA was used as a template for quantitative real-time PCR analysis.

Sequences for primers of insulin related genes (InsR and DPP-IV), anti-apoptosis pathway (AKT, PKCα, PKA, PI3K) and β-Actin were obtained from Genbank and NCBI. Primers were designed with Primer Express software and synthesized at Shangong (Shanghai, China) (Table 6). For qRT-PCR reactions, 25 μL mixtures were made by using SYBR® Premix Ex TaqTM II (DRR820A, Takara) containing 12.5 μL SYBR® Premix Ex TaqTM II, 1.0μL of forward and 1.0 μL of reverse primer, 8.5 μL ribonuclease(RNase)-free water and 2 μL cDNA. The real-time PCR reaction conditions were consisted of 3 min at 95 °C (first segment, one cycle), 10 s at 95 °C and 30 s at Tm of a specific primer pair (second segment, 44 cycles) followed by 10 s at 95 °C, and 72 °C for 10 s (dissociation curve segment) using Thermal Cycler (C1000, BIO RAD, USA). The results from the qRT-PCR were analyzed with 2^-ΔΔCT^ assay and β-Actin was used as an internal control gene. All data were presented in terms of relative mRNA, expressed as Means ±SE.

**Table 6:** Primer used in this study for qRT-PCR

Primers	Sequences(5′-3′)	Fragment Size(bp)	Tm (°C)
InsR	ACCTGTACCCTGGAGAGGTGCCCTCAATGACCGAGCAG	91	60
AKT	GTGGCAAGATGTGTATGAGACTGGCTGAGTAGGAGAACTG	195	58
PKCα	TGGCGTCCTGTTGTATGAAATCGTCTTCATCTTCACCATCA	245	58
PKA	TGTCAACTTCCCGTTCCTGGGCTCACTGAACCTCCCAATCC	131	60
PI_3_K	TGCTACCTTATGCCTGTCTATCCATGTTCTTGTCCTTGAGCCACTG	159	56
DPP-IV	TCAGAACTATTCCGTGATGGCGGCCTGTGGCACTCGTTTCAATA	100	60
β-actin	AGGGAAATCGTGCGTGACATGAACCGCTCATTGCCGATAG	150	58

### Statistical analysis

Data were expressed as mean ± SD of the number of animals used in each experiment. Statistical analysis was performed using two-way ANOVA and Tukey post hoc test. Values of *p* < 0.05 were considered statistically significant.

## Abbreviations

Alb: Albumin
ALP: Alkaline phosphatase
ALT: Alanine aminotransferase
AST: Aspartate aminotransferase
FBG: Fasting blood glucose
GLP-1: Glucagon-like peptide-1
GSH: Glutathione
HDL-C: High-density lipoprotein cholesterol
INS: Insulin
IR: Insulin resistance
LDL-C: Low-density lipoprotein cholesterol
MDA: Malondialdehyde
OGTT: Oral glucose tolerance tests
qRT-PCR: Quantitative real-time PCR
SOD: Superoxide dismutase
STZ: Streptozotocin
T2DM: Type 2 diabetes mellitus
T-CHO: Total cholesterol
TG: Triglyceride
TP: Total protein
2HPBG: Two hours postprandial blood glucose
